# Curcumin-Loaded Hydrophobic Surface-Modified Hydroxyapatite as an Antioxidant for Sarcopenia Prevention

**DOI:** 10.3390/antiox10040616

**Published:** 2021-04-16

**Authors:** Ya-Jyun Liang, I-Hsuan Yang, Yi-Wen Lin, Jhih-Ni Lin, Chang-Chin Wu, Chih-Yung Chiang, Kun-Hung Lai, Feng-Huei Lin

**Affiliations:** 1 Institute of Biomedical Engineering, College of Medicine and College of Engineering, National Taiwan University, Taipei 10617, Taiwan; d04548016@ntu.edu.tw (Y.-J.L.); f07528014@ntu.edu.tw (I.-H.Y.); d06548012@ntu.edu.tw (Y.-W.L.); d08548003@ntu.edu.tw (J.-N.L.); 01259@km.eck.org.tw (C.-Y.C.); 12044@km.eck.org.tw (K.-H.L.); 2 Department of Orthopedics, En Chu Kong Hospital, New Taipei City 23702, Taiwan; 00709@km.eck.org.tw; 3 Division of Biomedical Engineering and Nanomedicine Research, National Health Research Institutes, No. 35, Keyan Road, Zhunan, Miaoli County 35053, Taiwan

**Keywords:** sarcopenia, antioxidant, curcumin, hydroxyapatite, drug delivery

## Abstract

Oxidative stress and later-induced chronic inflammation have been reported to play an important role on the progression of sarcopenia. Current treatments for sarcopenia are mainly administered to patients whom sarcopenia already developed. However, there has been no promising results shown in therapy. Therefore, the development of therapeutic and preventive strategies against sarcopenia would be necessary. Curcumin is a traditional medicine that possesses anti-inflammatory and antioxidative properties. In the present study, hydroxyapatite was subjected to hydrophobic surface modifications for curcumin loading (Cur-SHAP). It was, subsequently, utilized for delivery to the patient’s body via intramuscular injection in order to achieve constant release for more than 2 weeks, preventing the progression of the sarcopenia or even leading to recovery from the early stage of the illness. According to the results of WST-1, LIVE/DEAD, DCFDA, and gene expression assays, Cur-SHAP exhibited good biocompatibility and showed great antioxidant/anti-inflammatory effects through the endocytic pathway. The results of the animal studies showed that the muscle endurance, grip strength, and fat/lean mass ratio were all improved in Cur-SHAP-treated rats from LPS-induced sarcopenia. In summary, we successfully synthesized hydrophobic surface modification hydroxyapatite for curcumin loading (Cur-SHAP) and drug delivery via the IM route. The LPS-induced sarcopenia rats were able to recover from disease after the Cur-SHAP treatment.

## 1. Introduction

Sarcopenia is a condition characterized by progressive loss of muscle endurance muscle mass and muscle strength. Patients with sarcopenia are prone to fractures and having low mobility. In addition, they display important comorbidities, such as anemia, heart failure, and osteoporosis, which often limit their engagement in physical activities [1]. According to previous studies, muscle dysfunction, which has been linked to sarcopenia, and the loss of muscle mass involves not only contractile impairment but also metabolic and endocrine abnormalities. These abnormalities, including chronic low-grade systemic chronic inflammation, systemic inflammation, and oxidative stress, negatively affect the metabolism and immune system of the host [2]. Aging has been associated with chronic inflammation; as the plasma levels of pro-inflammatory mediators increase, sarcopenia is induced [3].

The following progressive stages of sarcopenia: pre-sarcopenia, sarcopenia, and severe sarcopenia have been proposed by the European Working Group on Sarcopenia in Older People (EWGSOP). Pre-sarcopenia is characterized by muscle mass lower without reductions in physical performance or muscle strength [4]. Accordingly, a person with pre-sarcopenia may fully recover if adequate treatment is provided. The development of therapeutic and preventive strategies against sarcopenia and muscle disorders is currently an epidemiological need [5,6]. In the present study, we combined curcumin with a biodegradable ceramic and administered this formulation via the intramuscular (IM) route to achieve controlled curcumin release. The aim was to either prevent the worsening of pre-sarcopenia or improve recovery from pre-sarcopenia.

**Figure 1:**
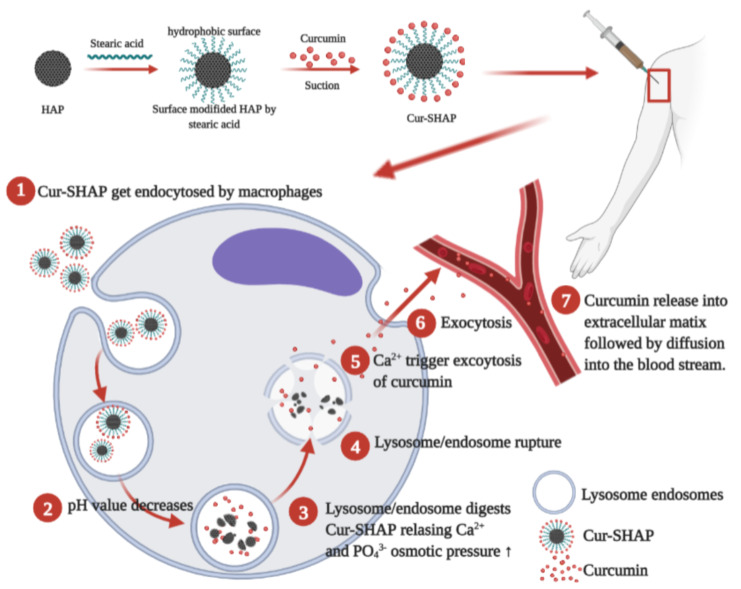
The bioactive Cur-SHAP particle and its in vivo delivery via the intramuscular (IM) route. The sequence of steps used to synthesize Cur-SHAP particles is shown. The method employed to administer the Cur-SHAP particles via IM is indicated, along with the steps and mechanisms involved in the delivery of curcumin to the blood circulation system.

Curcumin is a component of the herb turmeric that has been employed as part of traditional medicines [7]. Curcumin has many medicinal characteristics, such as anti-inflammatory and antioxidant activities [8]. Many studies have reported that curcumin may prevent loss of skeletal muscle mass and may stimulate muscle regeneration after injury [9]. According to prior studies, NFкB inhibition is one of the mechanisms whereby curcumin exerts its anti-inflammatory effects. Curcumin supplementation also promotes muscle growth and recovery [10]. Despite extensive research and development, the reports of limited clinical efficacy and low bioavailability of curcumin have been attributed to its poor absorption, rapid metabolism, and fabrication as an oral drug, which might rapidly degrade following digestion [11]. Therefore, to increase its bio-efficacy, researchers have made attempts to combine curcumin with carriers to develop drug delivery systems that enable its constant release. However, the clinical requirement of constant release for at least 2 weeks has not been achieved. Although hydroxyapatite (HAP) release has not been easily controlled in previous studies, it has been widely used in drug delivery systems [12,13]. In this study, a porous HAP was synthesized and subjected to a series of hydrophobic surface modifications with stearic acid (SHAP) to enable curcumin loading via physical adsorption [13]. We aimed to test the hypothesis that curcumin-loaded SHAP (Cur-SHAP) delivered via the IM route remains in muscle tissues and is gradually assimilated by defense cells such as macrophages via endocytosis. Additionally, we aimed to determine if the endosome with Cur-SHAP later coalesces with a lysosome to form an endosome/lysosome hybrid, enabling full dissolution of HAP. Once HAP is dissolved, the endosome/lysosome hybrid would be broken down by the osmotic pressure induced by increased Ca^2+^ and PO_3_^4−^ concentration in the hybridsomes. Through this mechanism, curcumin would be released into the cytoplasm and, subsequently, the extracellular space due to high Ca^2+^ levels [12]. Thereafter, curcumin would enter the vascular circulation by diffusion and be transported throughout the body. Of note, the biodegraded products of Cur-SHAP are Ca^2+^, PO_3_^4−^, and curcumin. Among these, the calcium ions could improve muscle function and curcumin could be the bioingredient that exerts antioxidant and anti-inflammatory effects to prevent sarcopenia or relieve its symptoms [14]. The overall experimental design is displayed in Figure 1.

## 2. Materials and Methods

### 2.1. Cur-SHAP Preparation

HAP was synthesized via a co-precipitation method. Briefly, a 0.5 M calcium hydroxide (Ca(OH)_2_, 1305-62-0, Sigma-Aldrich, St. Louis, MO, USA) solution was prepared by dissolving 3.86 g Ca(OH)_2_ in 100 mL ddH_2_O and incubating the resulting solution at 85 °C overnight. Thereafter, a stoichiometric amount (Ca/p molar ration = 1.67) of 100 mL 0.3 M phosphoric acid (H_3_PO_4_, 7664-38-2, Sigma-Aldrich, St. Louis, MO, USA) was added drop-wise at a rate of approximately 3 mL/min into the previous 0.5 M Ca(OH)_2_ solution. Baking powder (15 g) was added to serve as a frothing agent to make capable porosity and pore size. The mixture was magnetically stirred for 2 h and incubated at 85 °C for 20 h. After incubation, the precipitated particles collected and washed with methanol 3 times followed by deionized water. The particles were calcined at 800 °C to remove the baking powder and obtain the HAP particles.

0.1 g Stearic acid (Sigma-Aldrich, St. Louis, MO, USA) was stirred in 200 mL of ddH_2_O and heated at a 90 °C without organic solvents. Approximately 5.0 g of HAP powder was added and stirred for 24 h at a low temperature without organic solvents. The mixture was collected and centrifuged at 6000 rpm for 10 min, washed with ddH_2_O 3 times, and lyophilized. The particles collected were surface-modified HAP (SHAP).

Approximately 200 mg of curcumin (Sigma-Aldrich, St. Louis, USA) was added to 10 mL of ddH_2_O and mixed with 500 mg of SHAP particles. A vacuum system was used to assist the transport of curcumin stuck in the pores of SHAP. The particles were collected and centrifugation at 6000 rpm for 10 min, washed with ddH_2_O 3 times, and lyophilized. The particles collected were curcumin-loaded HAP (Cur-SHAP).

### 2.2. Material Characterization

The functional groups were analyzed by Fourier transform infrared spectroscopy (FTIR, JASCO 410, Tokyo, Japan) in the scanning range of 400–4000 cm^−1^ at a scanning rate of 400 nm/min.

The crystal structure was identified using an X-ray diffractometer (XRD, Rigaku Geigerflex, Tokyo, Japan). The XRD patterns were obtained at 30 kV and 15 mA, within the range of 10–70° at a scanning rate of 1°/min [15].

The morphology of the synthesized particles was examined using scanning electron microscopy (SEM, Philips XL30, Amsterdam, The Netherlands), with a voltage of 15 kV. Particles were mounted on the sample stage of the SEM and coated with a platinum-sputtered coating [16].

The particle sizes and zeta potential were detected by dynamic light scattering (DLS, Malvern, Worcestershire, UK) at 25 °C. The zeta potential was determined with electrophoretic mobility at pH 7.4. The sample was dispersed in ddH_2_O to measure the mean size.

The pore size distribution and specific surface area were analysis by Brunauer, Emmett, and Teller (BET; Micromeritics ASAP2010, Norcross, GA, USA) using nitrogen gas adsorption–desorption isotherms.

### 2.3. Curcumin Loading Efficiency and Release Profile

Thermogravimetric analysis (TGA, CAMCOR, Dynamic Q500) was used to determine the amount of curcumin in Cur-SHAP. The sample (5 mg) was heated at a rate of 10 °C/min from 20 °C to 800 °C under nitrogen flux. The weight lost at different temperatures was recorded using a computer system. The weight lost at the volatilization temperature of curcumin was used to determine the amount of curcumin in Cur-SHAP [17].

In vitro curcumin release was examined in a phosphate buffer solution (PBS) at pH 3 and 7 to mimic the environment of the lysosome/endosome hybrid and the physiological environment, respectively. Briefly, 2 mg Cur-SHAP was immersed in 50 mL PBS and the temperature was maintained at 37 °C for certain periods (supernatants were used to quantify the release in the first 2, 6, 12, and 24 h; supernatants were collected every 2 d over a period of 11 d). The supernatants were dissolved in DMSO (DMSO/supernatants = 9:1) for further analysis by UV–VIS spectrophotometry (JASCO V-670, Tokyo, Japan) at a wavelength of 430 nm.

To confirm that curcumin is released from Cur-SHAP in a relatively short period in the endosome/lysosome hybrids, we seeded RAW-264.7 (Bioresource Collection and Research Center, FIRDI, Hsinchu, Taiwan) macrophages on a 10^6^/10 cm dish for culture with 30 mg of Cur-SHAP particles. RAW-264.7 was cultured in 30 mL of PBS with Cur-SHAP for 3 d, because of the interference of the medium on UV–VIS. The supernatants were collected and centrifuged to remove any interference. The supernatants were dissolved in DMSO (DMSO/supernatants = 9:1) for further analysis by UV–VIS spectrophotometry at a wavelength of 430 nm.

### 2.4. Internalization of Cur-SHAP by RAW-264.7 Macrophages

RAW-264.7 cells were cultured on a 24-well plate at a density of 10^5^ cells/well for 24 h. The cells were then treated with Cur-SHAP (0.3 mg/mL) for 24 h, collected by centrifugation, washed 2 times with PBS, fixed with 2.5% glutaraldehyde for 24 h, and post-fixed in 1% osmium tetroxide solution for 2 h. The cells were then rinsed 3 times with 0.2 M PBS buffer and dehydrated in a graded series of ethanol. To observe the internalization of the particles, we captured images using TEM (Jeol, JEM-1200EX II, Tokyo, Japan) operated at 100 kV.

### 2.5. Evaluation of Cell Viability and Cytotoxicity

The viability of cells treated with Cur-SHAP was evaluated using the water-soluble tetrazolium (WST-1, Takara) assay. C2C12 myoblasts (Bioresource Collection and Research Center, Hsinchu, Taiwan) were passaged in growth medium containing Dulbecco’s modified Eagle’s medium (DMEM; Gibco, MD, USA), 1% antibiotic–antimycotic (Gibco), and 10% fetal bovine serum (FBS; Hyclone, USA), and then incubated at 37 °C in a humidified 5% CO_2_ incubator (Nozaki, Nikai, Okabe, Nagahama, and Eto, 2016). Briefly, C2C12 myoblasts were seeded in 96-well plates at a density of 10^4^ cells/well and cultured for 1 d to full adhesion. An extract medium was prepared using 0.2 g/mL HAP and Cur-SHAP and incubated for 1 day. The extracted supernatant was collected for subsequent experiments. Aluminum oxide (Al_2_O_3_, Sigma-Aldrich) and zinc diethyldithiocarbamate (ZDEC, Sigma-Aldrich) were used as negative and positive controls, respectively. The extracted medium was cultured with C2C12 myoblasts for 24 h. Thereafter, 100 μL WST-1 solution was added to the wells and incubated for 2 h. The absorbance values of each well were measured at 450 nm using an ELISA plate reader (Molecular Devices) [18,19].

Cytotoxicity was evaluated using the LIVE/DEAD staining kit (L3224, Invitrogen, USA) according to the manufacturer’s instructions [19]. C2C12 myoblasts were cultured in a 24-well plate at a density of 3 × 10^4^ cells/well and incubated for 24 h for full adhesion. Thereafter, 0.1 mg/mL HAP and Cur-SHAP were added into each well of the culture plate and incubated for 24 h. LIVE/DEAD staining was observed using a fluorescence microscope (IX81, Olympus).

### 2.6. Detection of Cellular ROS Generation

The ability of Cur-SHAP to suppress LPS-induced ROS generation was measured using a DCFDA-cellular ROS assay kit (ab113851, Abcam). Briefly, C2C12 myoblasts were cultured in 96-well plates at a density of 10^4^ cells/well and incubated for 24 h. After the medium was supplemented with 1 mg/mL of Cur-SHAP for 24 h, 1 μg/mL LPS (L2880, Sigma) was added to induce ROS generation for 24 h. The medium was then removed, washed with PBS 2 times, and cultured in medium with 25 μM DCFDA reagent for 45 min at 37 °C. The fluorescence signal was detected by multimode microplate readers (Molecular Devices, SpectraMax i3x, USA) with emission and excitation wavelengths of 535 nm and 485 nm, respectively.

### 2.7. RNA Extraction and Gene Expression

C2C12 cells (2 × 10^5^ cells/well) were cultured in 6-well plates for 24 h. Thereafter, the medium was changed with fresh DMEM with 100 µg/mL Cur-SHAP. A blank medium was employed as the control group. After 24 h of treatment, fresh medium was replaced with medium containing 1 µg/mL LPS to stimulate inflammation. After 4 h of treatment, the C2C12 cells were collected, the total RNA was extracted with Direct-zol™ RNA MiniPrep Kits (Zymo Research, Irvine, CA, USA). After that, RNA reverse transcription to cDNA using SuperScript™ III reverse transcriptase (Thermo Fisher). For qPCR analysis, cDNA was mixed with primers and SYBR Green Master Mix (Thermo Fisher). The primers used in the analysis are summarized in Table S1. Relative expression levels of IL-6, TNF-α, and Atrogin-1 gene were normalized to that of GAPDH. The intensity was detected and recorded by LightCycler^®^ 480 Instrument (Roche Diagnostics Nederland BV, The Netherlands).

### 2.8. Animal Model of LPS-Induced Sarcopenia

Male Sprague Dawley rats (12 months of age, 500–600 g) were purchased from Bio LASCO Taiwan Co., Ltd. All animal experiments were performed in accordance with the guidelines of the National Taiwan University, College of Medicine, Institutional Animal Care and Use Committee (IACUC, no. 20190096).

The experiments were conducted over a period of 2 months. LPS (L2880, Sigma-Aldrich) was dissolved in normal saline before injection. Sarcopenia with muscle injury was induced via an intraperitoneal (IP) injection of LPS twice per week, as described previously [20,21]. The Sprague Dawley rats were randomly divided into 3 groups of 6: (1) control group, IP injected with 1 mL sterile saline; (2) LPS group, IP injected with LPS (150 μg/kg BW) twice per week to induce sarcopenia; and (3) LPS-Cur-SHAP group, IM injected with 150 mg/kg Cur-SHAP at 0, 14, 28, and 42 d before IP injection. The rats were subjected to treadmill and grip strength tests before being killed. After killing, rat muscle was harvested for MRI examination, and whole blood was collected for safety evaluation.

### 2.9. Treadmill and Grip Strength Tests

The muscle endurance of Sprague Dawley rats was evaluated using the treadmill test (Exer-6M Treadmill; Columbus Instruments, OH, USA). For 1 week, all rats were trained to run by running on a treadmill for 30 min at a starting speed of 2 m·min^−1^ and ending at 20 m·min^−1^ (2 m·min^−1^ increase). The bottom of the treadmill was considered a low performance section and was equipped with an electrical stimulation component. Muscle endurance was determined by measuring the time spent by the rats in the low performance section of the treadmill during the 30 min test [22].

The muscle function of rats was evaluated using the grip strength test and hanging grip test. Grip strength was measured using a grip strength meter (Columbus Instruments, Columbus, OH, USA), according to a previous report [23]. The paws of rats were placed on a wire grid, and their tail was pulled backward; the maximum strength of the grip was recorded according to the strongest grip of the rat on the wire grid. Each rat was tested 6 times at 5 min intervals. The results are expressed in grams.

### 2.10. Fat-to-Muscle Ratio Determination by MRI 

MRI was used to assess muscle morphology and measure the fat-to-lean mass ratio. After rats were sacrificed, their hind legs were harvested and subsequently mounted onto a Bruker Biospec 7T MRI (Bruker Corporation, Billerica, MA, USA). The framed parameters were set as TE = 3.776 ms, TR = 25 ms, FA = 30°, NA = 1, MTX = 256 × 256 × 128, and resolution = 110 × 110 × 400 μm. Fat-to-lean mass ratio was analyzed using ImageJ software.

### 2.11. Blood Biochemical Analysis

The safety of Cur-SHAP in vivo was evaluated via serological analysis. After animals were killed, blood was collected via cardiac puncture. Thereafter, serum was obtained via centrifugation at 1300× *g* at 4 °C for 15 min. The collected serum was stored at −80 °C. For the biochemical tests, total protein (TP), creatine kinase (CK), lactate dehydrogenase (LDH), calcium (Ca), and alanine aminotransferase (ALT) in serum were measured. Serum analysis was performed by the National Taiwan University Veterinary Hospital, Taiwan. Reference: Charles River Laboratories, CD^®^ IGS Rat Model Information Sheet [16,24].

### 2.12. Statistical Analysis

Statistical data are expressed as mean ± standard deviation (SD). Statistical analysis was performed using one-way ANOVA. Differences were considered significant at a *p*-value of less than 0.05. (*p* < 0.05, *; *p* < 0.01, **; *p* < 0.001, ***).

## 3. Results

### 3.1. Functional Groups and Crystal Structure Identification

**Figure 2:**
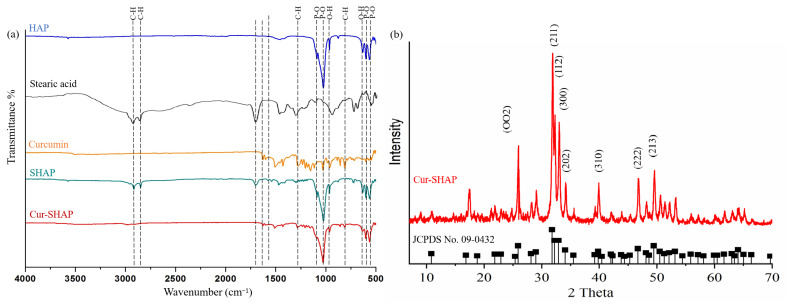
Identification of the functional groups and crystal structure of Cur-SHAP. (**a**) The absorption bands at 567, 604, and 3386 cm^−1^ were attributed to HAP. The absorption bands at 2924 and 2866 cm^−1^ were attributed to stearic acid. The absorption bands at 1510 and 1276 cm^−1^ were attributed to curcumin. Cur-SHAP displayed all the characteristic absorption bands contributed by HAP, stearic acid, and curcumin. (**b**) X-ray diffraction patterns of HAP, SHAP, and Cur-SHAP. All characteristic peaks matched those of the standard pattern of HAP in no. 09-0432 of the JCPDS card.

The FTIR spectra are presented in Figure 2a. The absorption bands at 3570 and 630 cm^−1^ were attributed to the O–H bond. The absorption bands at 1032 and 570 cm^−1^ for the P–O bonds were attributed to standard HAP. Finally, the absorption bands at 2924 and 2866 cm^−1^ for the C–H bonds were attributed to stearic acid. After HAP was surface-modified by stearic acid (SHAP), the characteristic absorption bands were found to be attributed to both HAP and stearic acid. The absorption bands at 1510 cm^−1^ and 1276 cm^−1^ for the C=C and C–H bonds were attributed to curcumin, respectively. The FTIR spectrum of Cur-SHAP displayed the characteristic absorption bands of SHAP and curcumin.

As displayed in the XRD patterns presented in Figure 2b, characteristic peaks, such as (002), (211), (300), (202), (310), (222), and (213), fully matched the standard XRD pattern of HAP with JCPDS card no. 09-0432. All characteristic peaks matched the XRD pattern of HAP, as previously determined.

### 3.2. Morphology, Particle Size, and Surface Area of Cur-SHAP

**Figure 3:**
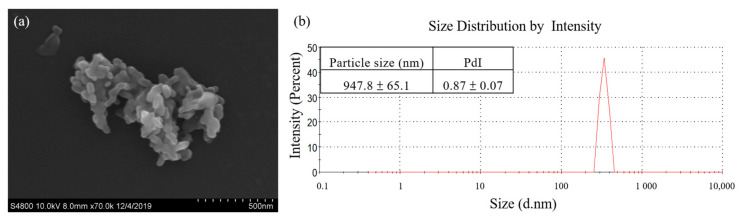
Morphology and particle size of Cur-SHAP. (**a**) SEM images of Cur-SHAP. The images reveal an open and interconnected porous structure. (**b**) Size distribution of Cur-SHAP analyzed by DLS. The results indicate that the Cur-SHAP particle size ranged from 900 to 1000 nm.

The morphology of Cur-SHAP was examined under SEM, as shown in Figure 3a. All grains showed a needle-like morphology and were 300–500 μm in length and 60–100 nm in width. Many pores were identified between the grains, leaving more surface area and space to accommodate curcumin.

The average particle size and distribution of Cur-SHAP determined using DLS are shown in Figure 3b and summarized in the attached table. The average particle size of Cur-SHAP was 947.8 nm, whereas the polydispersity index (PdI) was 0.87 due to aggregation. Such findings indicate that the size of Cur-SHAP ranged from 900 to 1000 nm, which is suitable for macrophage endocytosis.

The results of BET analysis are presented in Table 1. The average pore size and porosity of Cur-SHAP was 23.8691 nm; this value was higher than those of HAP prepared using the traditional method.

### 3.3. Determination of the Loading Efficiency of Curcumin

**Figure 4:**
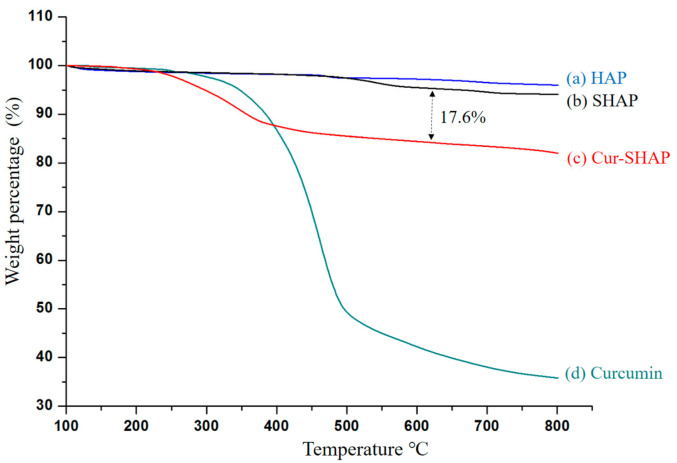
Curcumin loading efficiency analysis by TGA. The TGA curve of HAP, curcumin, SHAP, and Cur-SHAP is shown. Approximately 17.6% weight loss was due to the loss of curcumin.

TGA was used to evaluate the loading efficiency of curcumin, as shown in Figure 4. According to the HAP curve, as temperature increased from 25 to 800 °C, a minor loss in weight occurred. The curcumin curve also displayed a sharp weight loss at 307 °C, whereas the SHAP curve displayed negligible weight loss. The Cur-SHAP curve displayed a weight loss of approximately 17.6% due to the loss of curcumin.

### 3.4. Release Profile of Curcumin from Cur-SHAP and Examination of Cur-SHAP Particles in RAW-264.7 Macrophages

**Figure 5:**
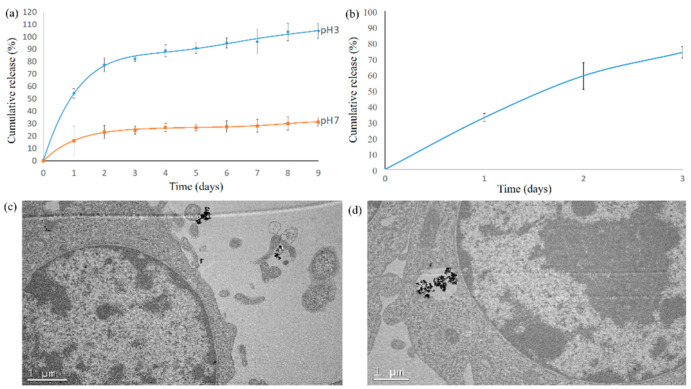
Release profile of curcumin. (**a**) The release profile of curcumin at pH 7 and 3, mimicking the conditions of the physiological environment and endosome/lysosome hybrid, respectively. (**b**) The drug release profile of Cur-SHAP co-cultured with RAW-264.7 macrophages; 70% of curcumin was consistently released within 2 d, presumably due to cellular activity. (**c**) TEM images of the interface of RAW-264.7 and Cur-SHAP particles prior to cellular uptake. (**d**) TEM images of Cur-SHAP particles transported to the lysosome, where the lysosome/endosome hybrid was inflated and ruptured due to the change in osmotic pressure.

The release profile of curcumin was evaluated by immersing the synthesized Cur-SHAP for a certain period in PBS solution (pH 7 or 3), as shown in Figure 5a. At the release profile at pH 7, which mimics physiological pH, an initial burst of 34% occurred within 1 h; further release of curcumin was not observed. At the release profile at pH 3, which mimics the acidic environment of the endosome/lysosome hybrid, the HAP carrier was dissolved, and curcumin was completely released from Cur-SHAP within 5 d. Figure 5b shows the curcumin release profile of Cur-SHAP co-cultured with RAW-264.7. Approximately 70% of curcumin was consistently released from Cur-SHAP within 2 d due to cellular activity. Such findings suggest that curcumin in Cur-SHAP was only released due to phagocytic activity and was not released in the normal physiological environment.

The TEM images presented in Figure 5 revealed the interface between RAW-264.7 cells and Cur-SHAP particles before endocytosis. Figure 5c shows that RAW-264.7 cells internalized the Cur-SHAP particles, which were enclosed in the endosome. As shown in Figure 5d, the Cur-SHAP particles were transported to the lysosomes and digested in the endosome/lysosome hybrid. Thereafter, due to the change in osmotic pressure, the hybrid was found to inflate and rupture. The process of cell uptake of Cur-SHAP particles presented in Figure 5 is conformable with our hypothesis that curcumin can be released via the phagocytic activities shown in Figure 1.

### 3.5. Cytotoxicity and Cell Viability Assays

**Figure 6:**
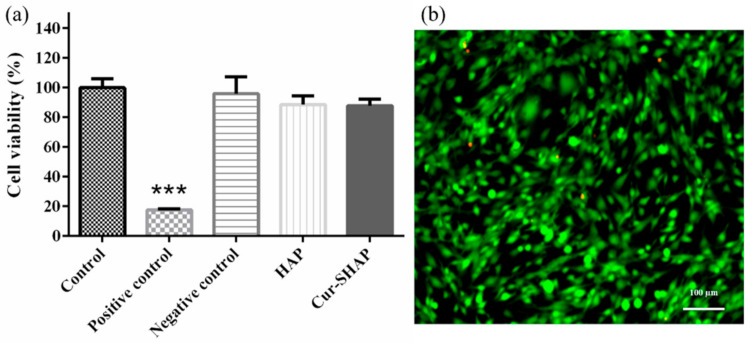
Cytotoxicity and cell viability assay. (**a**) Cell viability evaluation of Cur-SHAP using the WST-1 assay. (*n* = 6, *** *p* < 0.001 compared to the control). Zinc diethyldithiocarbamate and aluminum oxide served as the positive control and negative control, respectively. (**b**) Cytotoxicity assay of Cur-SHAP using LIVE/DEAD staining.

The effect of Cur-SHAP on cell viability was evaluated using the WST-1 assay (Figure 6a). The difference in cell viability between the control and Cur-SHAP groups was less than 10%. Therefore, Cur-SHAP was found to not affect the cell viability or the mitochondrial activity of C2C12 myoblasts.

The cytotoxic effect of Cur-SHAP was evaluated via LIVE/DEAD staining. As shown in Figure 6b, there was no cytotoxicity of Cur-SHAP groups. Accordingly, Cur-SHAP did not exhibit any cytotoxic effects on C2C12 myoblasts.

### 3.6. Antioxidant Effect of Cur-SHAP

**Figure 7:**
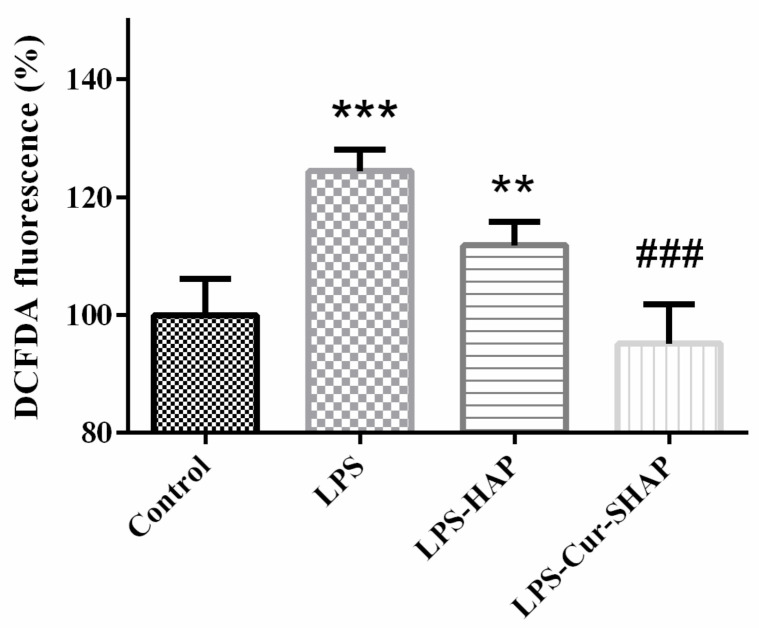
Cellular ROS generation measured using the DCFDA assay. These results confirm that Cur-SHAP exerts a good antioxidant effect. (*n* = 6, ** *p* < 0.01, *** *p* < 0.001 compared with control; *^###^ p* < 0.001 compared with LPS).

The antioxidant effect of Cur-SHAP was determined using the DCFDA assay, as shown in Figure 7. C2C12 cells cultured in DMEM were used as the control group. The ROS level in the Cur-SHAP group was lower than that in the LPS group. However, no significant difference in ROS levels was found between the control and Cur-SHAP groups. Cur-SHAP was thus confirmed to exhibit a good antioxidant effect.

### 3.7. Effect of Cur-SHAP on LPS-Induced Gene Expression

**Figure 8:**
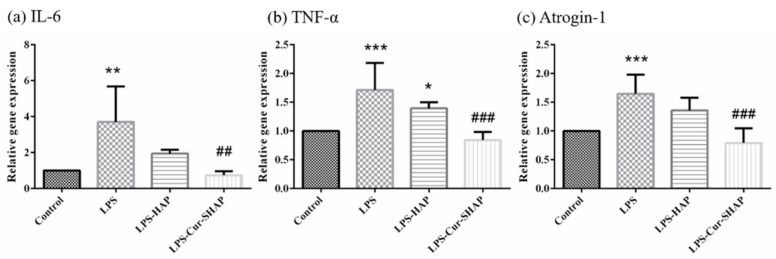
Gene expression of C2C12 after Cur-SHAP treatment and LPS induction for 24 h. Relative expression levels of (**a**) IL-6, (**b**) TNF-α, and (**c**) Atrogin-1 gene was measured by qPCR and normalized to that of GAPDH (*n* = 6, * *p* < 0.05, ** *p* < 0.01, *** *p* < 0.001 compared with control; *^##^ p* < 0.01, **^###^** *p* < 0.001 compared with LPS).

To explore the effect of Cur-SHAP on the properties of cells, we measured interleukin-6 (IL-6), tumor necrosis factor α (TNF-α), and Atrogin-1 via qPCR analysis. Accordingly, Cur-SHAP and LPS induction were found to affect the expression of IL-6, TNF-α, and Atrogin-1, as shown in Figure 8. The levels of IL-6, TNF-α, and Atrogin-1 in the LPS group were higher than those of the control group, whereas the levels of IL-6, TNF-α, and Atrogin-1 were significantly decreased in the Cur-SHAP group following LPS induction. The expression levels of IL-6 and TNF-α in the LPS group were increased by 11.35- and 1.64-fold, respectively, compared to those in the control group. However, after LPS induction, these levels were decreased by 6.22- and 0.64-fold, respectively, in the Cur-SHAP group, compared to those in the LPS group. The levels of IL6 and TNF-α were significantly decreased in the Cur-SHAP group after LPS induction. Cur-SHAP may thus decrease the gene expression level of IL-6 and TNF-α to inhibit LPS-induced inflammation.

### 3.8. Muscle Endurance Analysis Using the Treadmill Test and Grip Strength Measurement

**Figure 9:**
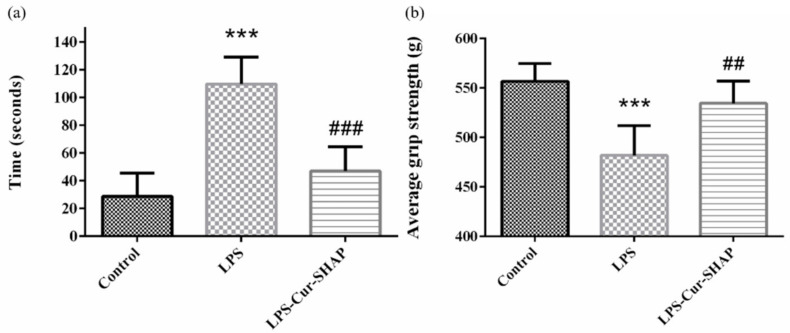
Muscle endurance and grip strength measurement. (**a**) Muscle endurance analysis using the treadmill test. (**b**) Average grip strength was measured using a grip strength meter (*n* = 6, *** *p* < 0.001 compared to the control group; ^##^ *p* < 0.01, ^###^ *p* < 0.001 compared to the LPS group). According to these results, muscle endurance and grip strength may be completely restored in rats with LPS-induced sarcopenia that have been treated with Cur-SHAP for 2 months.

A treadmill test was performed to evaluate the muscle endurance of rats with LPS-induced sarcopenia, as shown in Figure 9a. As per the 30-min treadmill test, the duration of low performance displayed by animals in the control, LPS, and LPS-Cur-SHAP groups was approximately 28, 109, and 47 s, respectively. Such findings suggest that muscle endurance could be effectively increased in rats with LPS-induced sarcopenia that had been administered Cur-SHAP via the IM route for 2 months.

The results of grip strength are presented in Figure 9b. The average grip strengths of rats in the control, LPS, and LPS-Cur-SHAP groups were 556.7, 481.9, and 534.5 g, respectively. Such findings suggest that grip strength may fully recover in rats with LPS-induced sarcopenia that have been treated with Cur-SHAP for 2 months.

### 3.9. The Ratio of Fat Mass to Lean Mass in Rat Muscle in Terms of MRI

**Figure 10:**
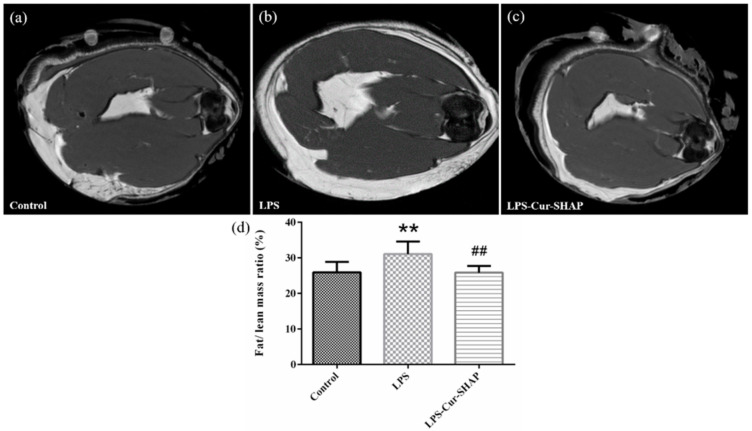
MRI images of the cross-section of the muscle in the (**a**) control group, (**b**) LPS group, and (**c**) LPS-Cur-SHAP group. (**d**) The ratio of fat and lean mass was calculated using image J (*n* = 6, ** *p* < 0.01 compared to the control; ^##^ *p* < 0.01 compared to the LPS group). The ratios of fat mass to lean mass in the muscles of the control, LPS, and LPS-Cur-SHAP groups were 25%, 31%, and 25%, respectively.

The cross-sections of rat muscles were examined using MRI (Figure 10). The white and black areas in the image respectively represent the fat mass and lean mass in the muscle. The ratio of fat mass to lean mass was calculated using the Image J software. The ratios of fat mass to lean mass in the muscles of the control, LPS, and LPS-Cur-SHAP groups were 25%, 31%, and 25%, respectively. There was no significant difference between the fat/lean mass ratio of the control group and the LPS-Cur-SHAP group.

### 3.10. Serological Analysis Determining Safety of Cur-SHAP In Vivo

The serum concentrations of TP, CK, LDH, Ca, and ALT is presented in Table 2. Values beyond the normal range (as obtained from the control group) are shown in red and are indicated as abnormal. In the LPS group, the values of CK, LDH, and ALT were beyond the normal range. However, in the LPS-Cur-SHAP group, the values of TP, CK, LDH, calcium, and ALT values were found to be within the normal range.

## 4. Discussion

In the present study, calcium hydroxide (Ca(OH)_2_), phosphoric acid (H_3_PO_4_), and baking powder (foaming agent) were employed to prepare porous HAP particles. By using the following materials, we were able to create a greater space and surface area for curcumin loading. Surface modification of HAP was then employed to improve the curcumin loading efficiency. Compared to the surface area of commercialized HAP, Cur-SHAP could have improved curcumin accommodation due to its high porosity and surface area (based on BET results; Table 1). The FTIR spectrum of Cur-SHAP displayed all absorption bands for HAP and curcumin (Figure 2). By assessing the morphology of Cur-SHAP, we recognized many pores between the grains, which left a greater surface area as well as space to accommodate curcumin (Figure 3) [13]. The mixing of surface-modified HAP with curcumin was achieved via a simple physical adsorption that enabled easy short-term drug release without constant release over a long period. Curcumin in the developed Cur-SHAP not only exhibited surface adsorption but was also entrapped in the lattice and enclosed between the grain boundary. The molar ratio of calcium to phosphorus in HAP, SHAP, and Cur-SHAP ranged from 1.40 to 1.67 (Table S2). The release of curcumin in the grain boundary and crystal lattice, combined with the process of cellular endocytosis, enabled the rapid dissolution of the Cur-SHAP particles in the endosome/lysosome hybrid. This was due to the low pH value in the hybrid environment. Curcumin escaped from the hybrid and was subsequently transported to the extracellular matrix for the delivery of curcumin throughout the entire body via the nearest blood vessel. Previously, a thermodynamic model was reported for many nanoparticle systems. This model revealed that the optimum cellular uptake particle size to endocytosis was 0.5–5 μm. According to the SEM and DLS analyses, the particle size of synthesized Cur-SHAP ranged from 0.5 to 1.5 μm, which is adequate for endocytosis (Figure 3).

To calculate the entrapment efficiency of curcumin in Cur-SHAP, we used the following formula: entrapment efficiency (%) = (the amount of curcumin in Cur-SHAP/total curcumin in the system) × 100%. Accordingly, the entrapment efficiency of curcumin in Cur-SHAP was identified to be approximately 54% (Figure 4). As a biomaterial, curcumin is widely used for bone repair, owing to its biocompatibility and nontoxicity to the human body (Figure 6).

Following the uptake of Cur-SHAP particles by cells, the particles were trapped in the endosome and would promptly merge with lysosomes to form endosome/lysosome hybrid at pH values of 3–5. The release profile of curcumin was determined by immersing Cur-SHAP particles in PBS solution (pH values of 3 and 7) to mimic the endosome/lysosome hybrid and physiological environment, respectively. The results of this experiment are shown in Figure 5a. Cur-SHAP immersed in PBS (pH = 3) was completely dissolved and 100% of curcumin was released within 4 d. Therefore, Cur-SHAP can be expected to dissolve in the acidic environment of the endosome/lysosome hybrid. Further, it can be expected to release all curcumin. However, when Cur-SHAP was immersed in PBS (pH = 7), an initial release burst of curcumin occurred in the first few hours due to physical adsorption; a plateau was subsequently observed. The curcumin release profile of Cur-SHAP co-cultured with RAW-264.7 is shown in Figure 5b. Curcumin in Cur-SHAP could only be released when phagocytic activity occurred. In fact, curcumin will not be released in the physiological environment owing to its retention in the HAP carrier.

In the present study, real-time PCR analysis was conducted to demonstrate the antioxidant and anti-inflammatory effects of Cur-SHAP against LPS-induced inflammation in C2C12 cells. As shown in Figure 8, the expression levels of IL-6, TNF-α, and Atrogin-1 in the LPS group were increased relative to the levels in the control group; however, these levels were significantly decreased in C2C12 cells treated with Cur-SHAP. Such findings indicate that Cur-SHAP dissolved and released curcumin to inhibit LPS-induced C2C12 injury, ultimately downregulating inflammatory gene expression.

Age-associated changes in the immune system, immune senescence, and chronic inflammation have been suggested to be major contributors to sarcopenia [2]. However, most studies used different substances to induce sarcopenia in young animals; this is because of limited availability and the very high costs associated with the use of aged animals [25]. LPS, one of the major molecular components on the outer membrane of Gram-negative bacteria, can cause a dysregulated inflammatory response [26]. Herein, muscle injury and body weight reduction were similar to the sarcopenia symptoms induced in the LPS-challenged rat model [27]. Meanwhile, the LPS-challenged rats showed lower muscle endurance and strength. The muscles of LPS-challenged rats mimicked those found in sarcopenia; characteristics include an increase in intermuscular adipose tissue infiltration and a decrease in the number and size of muscle fibers. Increased amounts of intermuscular adipose tissue correlate with the risk of cardiovascular disease. High serum LDH activity is a marker of cell damage [28,29] while serum CK is an indicator of muscle degradation. Because CK levels are highly sensitive to muscle injury, this level can be used as a tool to diagnose muscle damage [30,31]. Serum ALT activity is a reliable marker of liver disease and general health. Over the last decade, several studies have demonstrated that high serum ALT is associated with lower muscle mass, frailty, and sarcopenia [32]. The increase in LDH, CK, and ALT in sarcopenia-like rats could be detected via serological analysis (Table 2). Blood element analysis results indicated no sign of chronic toxicity in BSP-HAP (Table S3).

In skeletal muscle fibers, Ca^2+^ plays a crucial role in the excitation-contraction coupling process that induces an action potential in muscle fibers. In addition, Ca^2+^ is involved in numerous functions, such as myosin-actin cross bridging, protein synthesis, protein degradation, fiber type shifting. According to recent evidence, a dysregulation in Ca^2+^ is a common underlying phenomenon in the pathophysiology of muscles, such as sepsis, cachexia, sarcopenia, and heart failure [14]. Through endocytosis, curcumin might be released into the cytoplasm and eventually into the extracellular space due to a high level of Ca^2+^. Further, curcumin might enter the circulatory system via diffusion for transportation throughout the body. In previous studies, curcumin was found to play important roles in wound healing acceleration, endothelial cell proliferation, inducible nitric oxide stimulation, and anti-inflammatory and antioxidant activities [33,34]. Cur-SHAP may thus exert antioxidant effects and promote tissue repair in the recovery from LPS-induced muscle injury.

## 5. Conclusions

In this study, we successfully synthesized hydrophobic surface modification hydroxyapatite to enable curcumin loading (Cur-SHAP) via physical adsorption and drug delivery via the IM route. The characterized functional groups were identified and confirmed by FTIR; the results also aligned with those of previous reports. The size of the synthesized Cur-SHAP particles ranged from 500 to 1500 nm, which is an adequate size for cell uptake via the endocytic pathway. On the basis of the weight ratio, we found that the loading capacity of curcumin in Cur-SHAP was as high as 17.6%. Further, the release profile of curcumin from Cur-SHAP in vitro proved that curcumin would not be further released in the physiological environment after the initial burst. Instead, curcumin would be completely released and could escape from the endosome/lysosome hybrid. Cur-SHAP exhibited good antioxidant effects, thereby reducing oxidative stress and exerting anti-inflammatory effects. Herein, we aimed to administer one dose of Cur-SHAP particles via the IM route per month. According to the animal study, rats treated with Cur-SHAP via IM administration could effectively recover from LPS-induced sarcopenia, regardless of muscle endurance, grip strength, or fat/lean mass ratio measured using a treadmill, grip strength meter, and MRI, respectively. Following a long period of treatment with Cur-SHAP, its bio-safety in vivo was demonstrated via blood element analysis and serological analysis. Cur-SHAP delivered via the IM route has remarkable potential for application in sarcopenia therapy and sarcopenia prevention.

## Figures and Tables

**Table 1:** BET surface area and the porosity of Cur-SHAP. The BET surface area was 23.8691 m^2^/g while the Langmuir surface area was 38.8709 m^2^/g.

Sample	Result
**Cur-SHAP**	BET surface area: 23.8691 ± 0.2945 m^2^/g
	Correlation coefficient: 0.9993923
	Langmuir surface area: 38.8709 ± 0.9771 m^2^/g
	Correlation coefficient: 0.997482
	BJH adsorption average pore diameter: 25.7398 nm
	BJH desorption average pore diameter: 24.1160 nm

Brunauer–Emmett–Teller (BET) surface area analysis. Barrett–Joyner–Halenda (BJH) pore size analysis.

**Table 2:** Blood biochemical analysis of the serum concentration of total protein (TP), creatine kinase (CK), lactate dehydrogenase (LDH), calcium (Ca), and ALT (alanine aminotransferase). *n* = 6, * *p* < 0.05 compared to the control; ^#^ *p* < 0.05 compared to the LPS group. Reference: Charles River Laboratories, CD^®^ IGS Rat Model Information Sheet.

	Control	LPS	LPS-Cur-SHAP	Reference
Total protein (g/dL)	6.3 ± 0.2	6.4 ± 0.3	6.2 ± 0.2	6.6 ± 1.0
Creatine kinase (U/L)	115.4 ± 47.3	212.0 ± 62.1 *	132.5 ± 33.2 ^#^	131.3 ± 66.4
LDH (U/L)	364.0 ± 104.0	817.3 ± 105.9 *	402.1 ± 105.6 ^#^	500.0 ± 200.0
Calcium (mg/dL)	9.3 ± 0.8	9.2 ± 0.9	9.6 ± 0.4	9.5 ± 0.9
ALT (SGPT, U/L)	48.2 ± 17.0	110.6 ± 18.5 *	63.5 ±8.5 ^#^	56.72 ± 32.40

## Data Availability

The datasets used and/or analyzed in the current study are available from the corresponding author on reasonable request.

## Supplementary Materials

The following are available online at https://www.mdpi.com/article/10.3390/antiox10040616/s1, Table S1: Primers for real-time PCR. Table S2: Chemical composition of Cur-SHAP according to the atomic ratio and EDS analysis. Table S3: Safety of Cur-SHAP in vivo in terms of blood element analysis.
