# Supplementary material for: Curcumin-Loaded Hydrophobic Surface-Modified Hydroxyapatite as an Antioxidant for Sarcopenia Prevention
Source: Antioxidants (Basel). 2021 Apr 16;10(4):616. doi: 10.3390/antiox10040616 (PMC8073515; doi:10.3390/antiox10040616)
Supplement: Supplementary file 1 [file antioxidants-10-00616-s001.zip › supplementary-Supplementary data/supplementary-Supplementary data.pdf]

Table 1. Primers for Real-time PCR.

Primer	Sequence (5'→3')
**GAPDH forward**	TGGAAAGCTGTGGCGTGAT
**GAPDH reverse**	TGCTTCACCACCTTCTTGAT
**TNF-α forward**	GATCGGTCCCCAAAGGGATG
**TNF-α reverse**	TGAGGGTCTGGGCCATAGAA
**IL-6 forward**	GCTACCAAACTGGATATAATCAGGA
**IL-6 reverse**	CCAGGTAGCTATGGTACTCCAGAA
**Atrogin-1 forward**	GAGGCAGATTCGCAAGCGTTTGAT
**Atrogin-1 reverse**	TCCAGGAGAGAATGTGGCAGTGTT

Table S2. Chemical composition of HAP, SHAP and Cur-SHAP from EDS analysis by atomic ratio. The molar ratio of calcium to phosphorus in the Cur-SHAP that was 1.42.

Sample	C<br>Atomic %	O<br>Atomic %	P<br>Atomic %	Ca<br>Atomic %	Ca/P
HAP	17.92	53.78	10.62	17.69	1.6
SHAP	6.08	59.68	13.42	20.82	1.5
Cur-SHAP	51.01	38.70	4.24	6.05	1.4

Table S3. Safety of Cur-SHAP in vivo by blood element analysis. Reference: Charles River Laboratories, CD® IGS Rat Model Information Sheet. All results indicated no sign of chronic toxicity in Cur-SHAP.

	Control	LPS	LPS-BSP-HAP
RBC (M/μL)	9.23 ± 0.48	8.24 ± 0.21	8.96 ± 0.09
HGB (g/dL)	16.10 ± 0.56	14.25 ± 0.35	16.10 ± 0.14
HCT (%)	53.15 ± 2.89	46.10 ± 0.70	51.60 ± 0.70

MCV (fL)	57.60 ± 0.14	55.95 ± 0.63	57.55 ± 0.07
MCH (pg)	17.45 ± 0.35	17.30 ± 0.01	17.95 ± 0.07
MCHC (g/dL)	30.30 ± 0.56	30.90 ± 0.28	31.20 ± 0.14
RET (K/ μL)	249.10 ± 7.77	243.50 ± 6.92	197.60 ± 4.10
PLT (K/ μL)	1032.50 ± 7.77	1284.50 ± 54.85	1195.50 ± 24.74
WBC (K/ μL)	15.95 ± 0.62	19.99 ± 0.82	12.90 ± 0.26
NEUT (%)	11.90 ± 0.28	23.65 ± 17.04	22.80 ± 0.56
LYMPH (%)	76.35 ± 0.49	83.50 ± 7.77	70.50 ± 1.27
MONO (%)	5.80 ± 0.84	7.15 ± 0.21	5.30 ± 0.56
EO (%)	0.50 ± 0.01	1.25 ± 1.20	1.15 ± 0.07
BASO (%)	0.20 ± 0.01	0.15 ± 0.07	0.25 ± 0.07

RBC: red blood cell; HGB: hemoglobin; HCT: hematocrit; MCV: mean corpuscular volume: MCH: mean corpuscular hemoglobin; MCHC: mean corpuscular hemoglobin concentration; PLT: platelet; WBC: white blood cell; NEUT: neutrophil; LYMPH: lymphocyte; MONO: monocyte; EO: eosinophil; BASO: basophil.

	Control	LPS	LPS-Cur-SHAP
RBC (M/μL)	9.23 ± 0.48	8.24 ± 0.21	8.96 ± 0.09
HGB (g/dL)	16.10 ± 0.56	14.25 ± 0.35	16.10 ± 0.14
HCT (%)	53.15 ± 2.89	46.10 ± 0.70	51.60 ± 0.70
MCV (fL)	57.60 ± 0.14	55.95 ± 0.63	57.55 ± 0.07
MCH (pg)	17.45 ± 0.35	17.30 ± 0.01	17.95 ± 0.07
MCHC (g/dL)	30.30 ± 0.56	30.90 ± 0.28	31.20 ± 0.14
RET (K/ μL)	249.10 ± 7.77	243.50 ± 6.92	197.60 ± 4.10
PLT (K/ μL)	1032.50 ± 7.77	1284.50 ± 54.85	1195.50 ± 24.74
WBC (K/ μL)	15.95 ± 0.62	19.99 ± 0.82	12.90 ± 0.26
NEUT (%)	11.90 ± 0.28	23.65 ± 17.04	22.80 ± 0.56
LYMPH (%)	76.35 ± 0.49	83.50 ± 7.77	70.50 ± 1.27
MONO (%)	5.80 ± 0.84	7.15 ± 0.21	5.30 ± 0.56
EO (%)	0.50 ± 0.01	1.25 ± 1.20	1.15 ± 0.07
BASO (%)	0.20 ± 0.01	0.15 ± 0.07	0.25 ± 0.07

RBC: red blood cell; HGB: hemoglobin; HCT: hematocrit; MCV: mean corpuscular volume: MCH: mean corpuscular hemoglobin; MCHC: mean corpuscular hemoglobin concentration; PLT: platelet; WBC: white blood cell; NEUT: neutrophil; LYMPH: lymphocyte; MONO: monocyte; EO: eosinophil; BASO: basophil.
